# Metabolomics study of the therapeutic mechanism of *Schisandra Chinensis* lignans in diet-induced hyperlipidemia mice

**DOI:** 10.1186/s12944-017-0533-3

**Published:** 2017-08-01

**Authors:** Jing-Hui Sun, Xu Liu, Li-Xin Cong, He Li, Cheng-Yi Zhang, Jian-Guang Chen, Chun-Mei Wang

**Affiliations:** 1 College of Pharmacy, Beihua University, 3999 Binjiang East Road, Jilin, 132013 China; 2 Second Treatment Area of Senile Disease, First Affiliated Hospital of Changchun University of Traditional Chinese Medicine, 1478, Gongnong Road, Changchun, 130021 China

**Keywords:** *Schisandra chinensis* lignans, Hyperlipidemia, Metabolomics, RRLC-Q-TOF-MS, RT-PCR

## Abstract

**Background:**

Schisandra, a globally distributed plant, has been widely applied for the treatment of diseases such as hyperlipidemia, fatty liver and obesity in China. In the present work, a rapid resolution liquid chromatography coupled with quadruple-time-of-flight mass spectrometry (RRLC-Q-TOF-MS)-based metabolomics was conducted to investigate the intervention effect of *Schisandra chinensis* lignans (SCL) on hyperlipidemia mice induced by high-fat diet (HFD).

**Methods:**

Hyperlipidemia mice were orally administered with SCL (100 mg/kg) once a day for 4 weeks. Serum biochemistry assay of triglyceride (TG), total cholesterol (TC), low-density lipoprotein cholesterol (LDL-c) and high-density lipoprotein cholesterol (HDL-c) was conducted to confirm the treatment of SCL on lipid regulation. Metabolomics analysis on serum samples was carried out, and principal component analysis (PCA) and partial least squares-discriminant analysis (PLS-DA) were carried out for the pattern recognition and characteristic metabolites identification. The relative levels of critical regulatory factors of liver lipid metabolism, sterol regulatory element-binding proteins (SREBPs) and its related gene expressions were measured by quantitative real-time polymerase chain reaction (RT-PCR) for investigating the underlying mechanism.

**Results:**

Oral administration of SCL significantly decreased the serum levels of TC, TG and LDL-c and increased the serum level of HDL-c in the hyperlipidemia mice, and no effect of SCL on blood lipid levels was observed in control mice. Serum samples were scattered in the PCA scores plots in response to the control, HFD and SCL group. Totally, thirteen biomarkers were identified and nine of them were recovered to the normal levels after SCL treatment. Based on the Kyoto Encyclopedia of Genes and Genomes (KEGG) pathways analysis, the anti-hyperlipidemia mechanisms of SCL may be involved in the following metabolic pathways: tricarboxylic acid (TCA) cycle, synthesis of ketone body and cholesterol, choline metabolism and fatty acid metabolism. Meanwhile, SCL significantly inhibited the mRNA expression level of hepatic lipogenesis genes such as SREBP-1c, fatty acid synthase (FAS) and acetyl-CoA carboxylase (ACC), and decreased the mRNA expression of liver X receptor α (LXRα). Moreover, SCL also significantly decreased the expression level of SREBP-2 and 3-hydroxy-3-methylglutaryl coenzyme A reductase (HMGCR) in the liver of hyperlipidemia mice.

**Conclusion:**

Anti-hyperlipidemia effect of SCL was confirmed by both serum biochemistry and metabolomics analysis. The mechanism may be related to the down-regulation of LXRα/SREBP-1c/FAS/ACC and SREBP2/HMGCR signaling pathways.

## Background

Hyperlipidemia is a consternation of several serum lipoprotein abnormalities, such as increased triglyceride (TG), total cholesterol (TC), low-density lipoprotein cholesterol (LDL-c), very low-density lipoprotein cholesterol (VLDL-c), and decreased high-density lipoprotein cholesterol (HDL-c) in serum, and also as one of the major risk factors that cause arteriosclerosis, cerebral stroke, coronary heart disease, myocardial infarction, type 2 diabetes and renal failure [1–4]. Fibrates, statins, bile acid sequestrants, nicotinic acid and cholesterol absorption inhibitors are commonly used for the treatment of hyperlipidemia [5]. But unfortunately, most of these drugs generally have side or toxic effects, such as statin-induced myopathy and fibrate-induced rhabdomyolysis [6, 7]. Therefore, the research and development of safe and effective anti-hyperlipidemia drugs are particularly important.

Schisandra, a famous traditional medicinal material in China, is the dried ripe fruit of *Schisandra chinensis* (Turcz.) Baill., and widely used for various medicinal purposes, such as hepatoprotection, anti-inflammation, antioxidant property, antitumor activity, and resistance to insulin resistance [8–10]. The main components of Schisandra are lignans, and many research has shown that *Schisandra chinensis* lignans (SCL) has obvious anti-hyperlipidemia effect, such as reduced hepatic TG and TC levels, in mice with hypercholesterolemia produced by high-fat diet (HFD) containing cholesterol/bile salt, but the therapeutic mechanism was still unclear [11–13].

With the development of gas chromatography/mass spectrometer (GC/MS), liquid chromatography/mass spectrometer (LC/MS), nuclear magnetic resonance (NMR) and related analysis softwares, metabolomics has been widely used to investigate disease diagnosis, biomarker discovery, toxicology and pharmaceutical study by global assessment of living systems metabolites and dynamic responses to pathophysiological stress [14–16]. For hyperlipidemia, metabolomics is widely used in diagnosis, monitoring drug treatment and developing drugs [17–21]. In the present study, with regards to the multifacet mechanisms of hyperlipidemia and holistic effects of SCL, a RRLC-Q-TOF-MS-based metabolomic approach was developed to evaluate the anti-hyperlipidemia effect of SCL by analyzing the characteristics of mouse hyperlipidemia induced by HFD and the mechanism was also investigated by observing the related protein gene expression.

## Experimental materials

### Experimental animals and feed preparation

Forty eight male C57BL/6 mice, weighing 19 to 21 g, were provided by Beijing Weitonglihua Experimental Animal Technology Co. Ltd. [license number: SCXK (Beijing) 2015–0001, specific pathogen free (SPF)]. The mice were housed in separate cages, with an intake of about 5 g feed/mouse daily and access to water freely. The animal room was disinfected once a week, the temperature was controlled at about 22 °C, the humidity was about 50%, the natural diurnal cycle was maintained, the mouse pad was replaced strictly in accordance with the requirements once each three days, and the mice were weighed and recorded twice a week. The animal experiments were approved by the Institutional Animal Care and Use Committee (IACUC) of Beihua University with the permit number: CPBHU IACUC2015–007. All mouse experimental procedures were performed in accordance with the Regulations for the Administration of Affairs Concerning Experimental Animals approved by the State Council of People’s Republic of China.

Both the general diet and HFD for the mice were purchased from Changchun City Yisi Experimental Animal Technology Limited Liability Company. The general diet contained 5% fat, 53% carbohydrates, 23% protein, and 19% other components, and the high-fat diet contained 2% cholesterol, 0.5% sodium cholate, 10% lard, 5% sugar, and 82.5% of the general diet.

### Chemicals and materials

HPLC-grade methanol and acetonitrile were purchased from TEDIA (USA). All the reference standards were purchased from Sigma Corporation (St. Louis, Mo, USA). The water used in the experiments was collected from a Milli-Q Ultra-pure water system (Millipore, Billerica, USA). Other chemicals were of analytical grade.

### SCL preparation and its method

*Schisandra chinensis* was purchased from Jilin Province Jian City Schisandra Planting Base, and identified by Department of Pharmacognosy, College of Pharmacy, Beihua University as the dried ripe fruit of Schisandra [*Schisandra chinensis* (Turcz.) Baill.]. Schisandra was ground into powders, and the lignans in it were extracted by supercritical CO_2_ fluid extraction. The extraction procedures included molecular distillation (the distillation temperature: 110 °C, the feed rate: 65 L/h, and the vacuum degree: 0.7 Pa), the treatment with AB-8 macroporous resin (the total lignans flow: 1.52 mg/mL, the elution rate: 1 mL/min, the ethanol concentration: 95%, and the elution rate: 78.9%), and silica gel column chromatography [200–300-mesh silica gel and elution with 60:1 (V/V) petroleum ether-ethyl acetate]. Finally, the lignan component was obtained and the total lignanoids absorption spectrum was detected at 570 nm by ultraviolet spectrophotometry. The results showed that the extract purity was 93.5%. The extract could be adjusted a desired concentration by adding distilled water to it and kept at 4 °C for use.

## Methods

### Establishment of mouse hyperlipidemia model

Forty eight male mice were randomly divided into control group (*n* = 24) fed with the general diet and hyperlipidemia model (*n* = 24) fed with the HFD. Four weeks later, they were fasted for 12 h, but with free access to water, and then their blood samples were taken from the canthus (in the state of ether anesthesia) for the detection of TC and TG contents in the mice’s serum, which could confirm whether the establishment of hyperlipidemia model in mice was successful or not. Twenty four control mice were randomly divided into control group and control + SCL group. Twenty four hyperlipidemia mice were randomly divided into model group and model + SCL group. Mice in both the control group and model group were given an equal volume of distilled water, and those in the model + SCL group and control + SCL group were given SCL (100 mg/kg) intragastrically for 4 weeks successively.

After the end of the administration, the mice were fasted for 12 h, but with free access to water, then were anesthetized with ether and their blood samples were taken by eyeball removal, and the blood samples were centrifuged (at 3500 r/min and 4 °C, for 10 min) to separate the serum for use. Immediately the mice’s livers were removed, washed with precooling saline, dried with filter paper, and cryopreserved at −80 °C until analysis.

### Determination of serum lipids

Serum levels of TC, TG, LDL-c and HDL-c in serum of mice were assayed using commercially available kits (Nanjing Jiancheng Biotech Inc., Nanjing, China) and the specific procedures were strictly in line with the kit instructions.

### RRLC/MS analysis

100 μL of the serum were added with 1 mL methanol, which was shaken for 30 s by a vertex vibrator, and then centrifuged at 13000 r/min for 5 min to obtain the supernatants. The supernatants were filtered with 0.22 μm filter membranes and the filtered supernatants were kept at −4 °C for the analysis.

The rapid resolution liquid chromatography (Agilent 1200 RRLC, Santa Clara, CA, USA) system was equipped with a binary pump, a micro-degasser, an auto-plate sampler, and a thermostatically controlled column apartment. The mass spectrometer (Agilent 6520 Q-TOF MS, Santa Clara, CA, USA) was equipped with an electrospray interface and automatic calibration system.

The liquid chromatography separation was performed on an Agilent SB-C18 column (100 mm × 3.0 mm, 1.8 μm, 600 bar) at a temperature of 25 °C. 0.1% formic acid (v/v) and acetonitrile were used as the mobile phases A and B, respectively. The initial elution was 5% B, and the gradient elution was programmed as followed: 0–11 min (5%–61% B), 11–20 min (61%–95% B). The flow rate was 0.3 mL/min. The injected sample volume was 5 μL. This RRLC system was connected to Q-TOF mass spectrometer.

The Q-TOF-MS scan range was set at *m/z* 50–1000 in both negative and positive modes. The conditions of the ESI source were as follows: drying gas (N_2_) flow rate was 8.0 L/min, drying gas temperature was set at 350 °C, nebulizer was set as 30 psig, capillary voltage was 3500 V, fragmentor was 175 V, and skimmer was 65 V.

### Biomarkers data and statistical analysis

The analysis of RRLC/MS data was performed on Agilent software Masshunter (version B 03.01) and Mass Profiler Professional (version B 02.02). The parameters were set as follows: Experiment type: Unidentified, Organism: *Mus musculus*, Minimum absolute abundance: 2000 counts, Compound alignment: RT window = 0.1% + 0.15 min and Mass window = 5.00 ppm + 2.0 mDa, and Baseline Option: Z-Transform. After all the data are filtered and normalized, univariate data analysis was carried out using *t*-test and a one-way analysis of variance (ANOVA), with statistical significance set at *P* < 0.05. Multivariate data were analyzed using principal component analysis (PCA) and partial least squares-discriminant analysis (PLS-DA). PCA was applied to identify outliers and detect data grouping or separation trends, and it also produced an overview of the data set. In PCA, the samples are classified by score plots and the potential biomarkers are found by loading plots. PLS-DA, focused on the actual class discriminating variation of data compared to the unsupervised approach of PCA. The data were subjected to PLS-DA where to establish the model forecasting. The PLS-DA model was validated by describing R^2^Y and Q^2^ values, R^2^ describes how well the data could be mathematically reproduced by the training model, the closer the R^2^ and Q^2^ values to 1, the more stable the model was. For the identification of potential biomarkers, the following databases were used: HMDB [22], LIPID MAPS [23], and KEGG [24].

### RNA extraction and real-time reverse transcription polymerase chain reaction (RT-PCR)

The total RNA was extracted from mouse liver tissues using Trizol reagent (Invitrogen, USA) according to the manufacturer’s instruction to investigate effects of SCP on the expression of TG- and TC-related genes. The reverse transcription (RT) by total RNA(1 μg), oligo (15) dT, and reverse transcriptase was performed using RT kits (Promega, Beijing, China) in a final volume of 20 μL. PCR primer sequences were designed using primer 6.0 software and synthesized by Dingguo Changsheng Biotechnology Co. Ltd. (Beijing, China). The gene expression levels were analyzed in duplicate using a SYBR Green kit (Promega, Beijing, China) according to the manufacturer’s instruction on real-time PCR System (ABI 7500, Biometra, Germany). All the primer lengths and annealing temperatures are listed in Table 1. The ^ΔΔ^Ct method was used for the relative quantification. The relative gene expression was normalized to the GAPDH expression level, and the ratios were presented as arbitrary units.

**Table 1:** Primers used for quantitative real-time PCR

Genes	Primer sequences	Length
SREBP-1c	Forward 5′-GGAGACATCGCAAACAAGC-3’	273 bp
	Reverse 5′-GGTAGACAACAGCCGCATC-3’	
SREBP-2	Forward 5′-CACCCATACTCAGGCTCG-3’	133 bp
	Reverse 5′-GCTTCACAAAGACGCTCAA-3’	
FAS	Forward 5′-ATCGCCTATGGTTGTTG-3’	127 bp
	Reverse 5′-TCACGACTGGAGGTTCTA-3’	
ACC	Forward 5′-TATCCCAACTCTTCCCTG-3’	116 bp
	Reverse 5′-CCTTCACATAGCCTTTCTC-3’	
HMGCR	Forward 5′-CTTGACGCTCTTGTGGA-3’	259 bp
	Reverse 5′-CCCTTTGGGTTACGG-3’	
LXR	Forward 5′-CTCAATGCCTGATGTTTCTCC-3’	154 bp
	Reverse 5′-TGACTCCAACCCTATCCCTAA-3’	

### Statistical analysis

All data were expressed as mean ± standard deviation (SD) ($\overline{\mathrm{x}}$±s). “*n*” denotes the sample numbers in each group. SPSS software (version 19.0 for Windows) was used for the statistical analysis. *P* < 0.05 and *P* < 0.01 was considered to be statistically significant.

## Results

### Changes in blood TC and TG contents in mice on HFD for 4 weeks

**Fig. 1:**
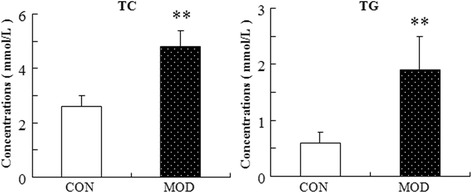
Comparison on serum TC and TG contents between control and model groups ($\overline{\mathrm{x}}$±s, *n* = 24). Note: CON: control group; MOD: model group. *: Compared with those in the control group, *P* < 0.01

As shown in Fig. 1, the content of TC and TG in the model group was significantly higher than that in the control group (*P* < 0.01), indicating that the HFD-induced hyperlipidemia model was successful.

### Effects of SCL on blood lipids in hyperlipidemic mice

**Fig. 2:**
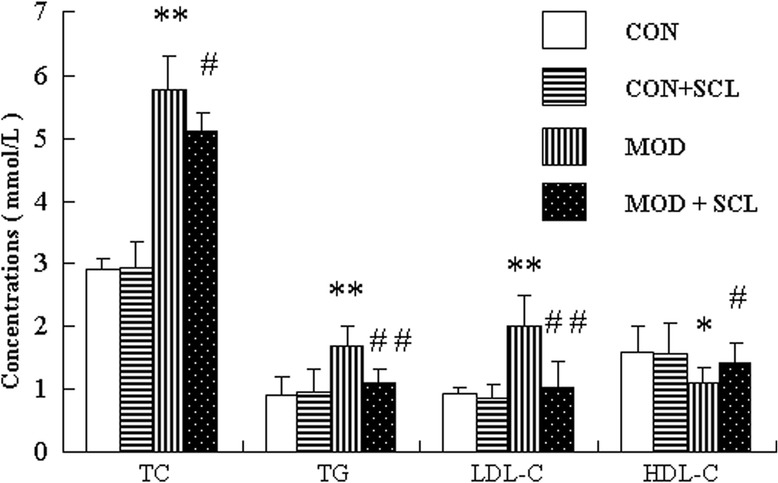
Comparison on serum TC, TG, LDL-c and HDL-c contents between groups ($\overline{\mathrm{x}}$±s, *n* = 12). Note: CON: control group; CON + SCL: control + SCL group; MOD: model group; MOD + SCL: model + SCL group. *: compared with those in the control group, *P* < 0.05, **: *P* < 0.01; ^#^: compared with those in the model group, *P* < 0.05, ^##^: *P* < 0.01

Four weeks after the administration, compared with those in the control group, serum TC, TG and LDL-c levels were significantly increased, and the level of HDL-c was significantly reduced in the model group (*P* < 0.05 or *P* < 0.01). Compared with those in the model group, serum TC, TG and LDL-c levels were significantly decreased, and the level of HDL-c were significantly increased in SCL group (*P* < 0.05 or *P* < 0.01). But the serum TC, TG, LDL-c and HDL-c levels between the control group and control + SCL group were not significantly different (*P* > 0.05). The results are shown in Fig. 2.

### Development of RRLC-Q-TOF-MS method for metabonomic analysis

**Fig. 3:**
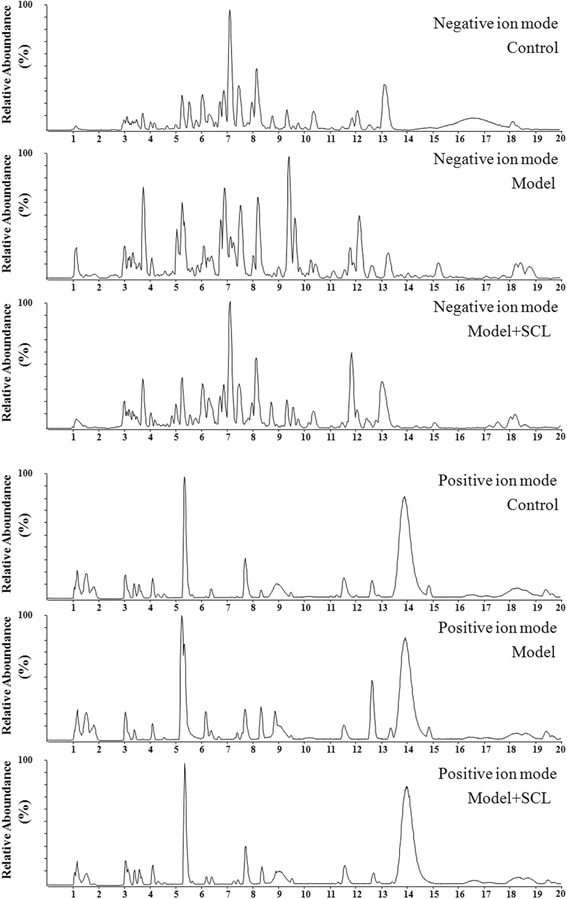
Base peak intensity (BPI) chromatograms obtained from the negative and positive ion RRLC-Q-TOF-MS analyses of control, model and model + SCL mouse

In this study, RRLC-Q-TOF-MS analysis was performed to acquire serum metabonomic profiles in positive and negative ion modes. Figure 3 shows the base peak intensity chromatograms (BPI) of serum sample from control, model and SCL mice in negative and positive ion modes. For the method validation study, a quality control (QC) sample was obtained by taking an aliquot of the same volume (50 μL) of all the serum samples in this study. The QC sample was measured after every six serum samples during all the LC-MS analyses. Five ions (*m/z* 233.2916, 264.1057, 297.6653, 374.1422 and 418.2501) in negative ion mode and five ions (*m/z* 203.0476, 246.2360, 274.2675, 302.2976 and 437.1807) in positive ion mode were selected. The extracted ion chromatograms (EIC) of the ten selected ions showed that the system stability (RSDs %) of retention times, m/z and peak areas were 0.07–0.21%, 0.0004–0.0009% and 6.2–9.7%, respectively. The results indicated that there was no significant difference among the every detection of QC sample and the system stability was excellent.

### Analysis of serum metabolite profiles

**Fig. 4:**
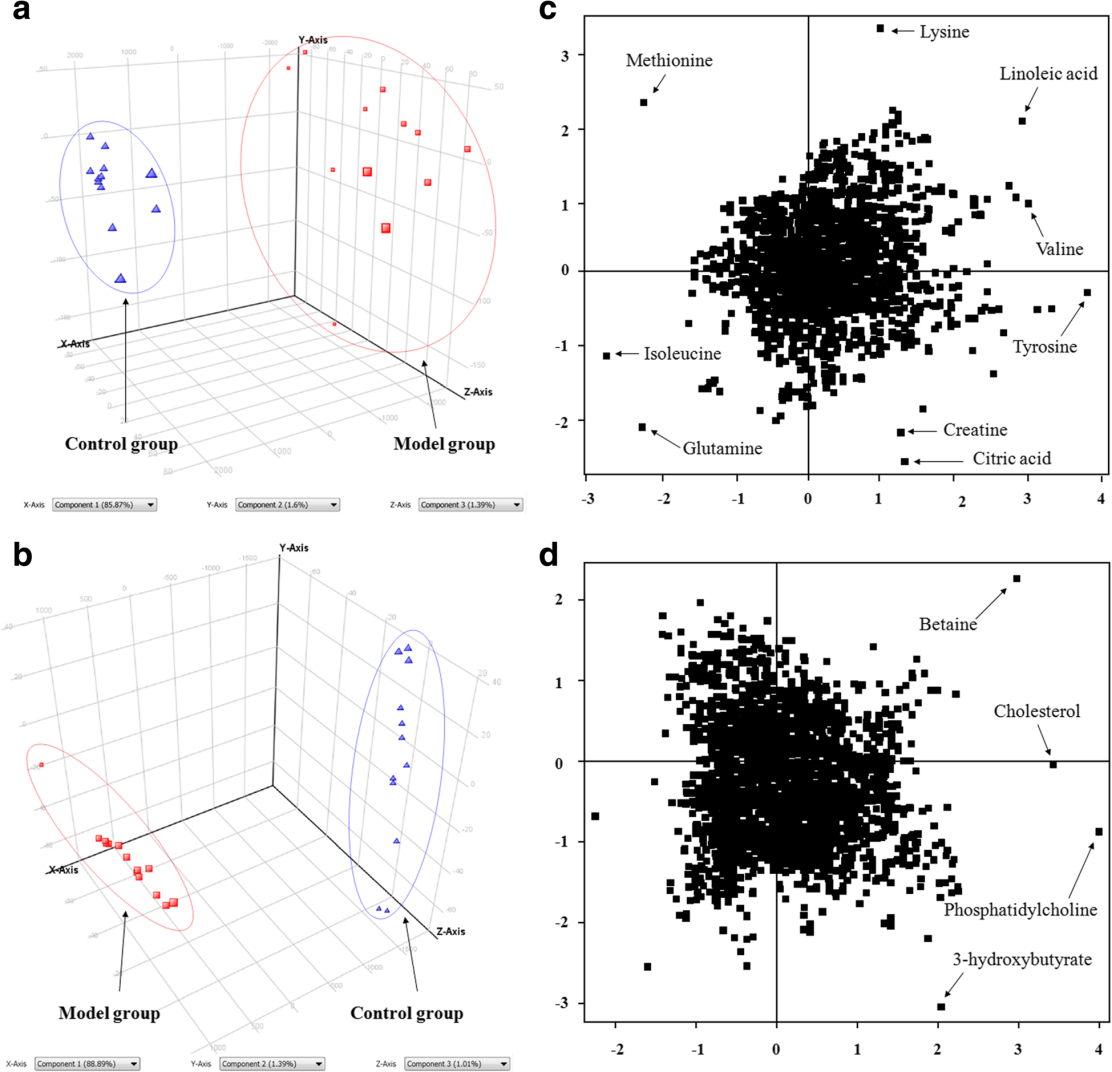
3D–PCA score plots of control and model group (: control group; : model group)in negative (**a**) and positive (**b**) ion mode; loading plots from the result of PCA of control and model group in negative (**c**) and positive (**d**) ion mode

**Fig. 5:**
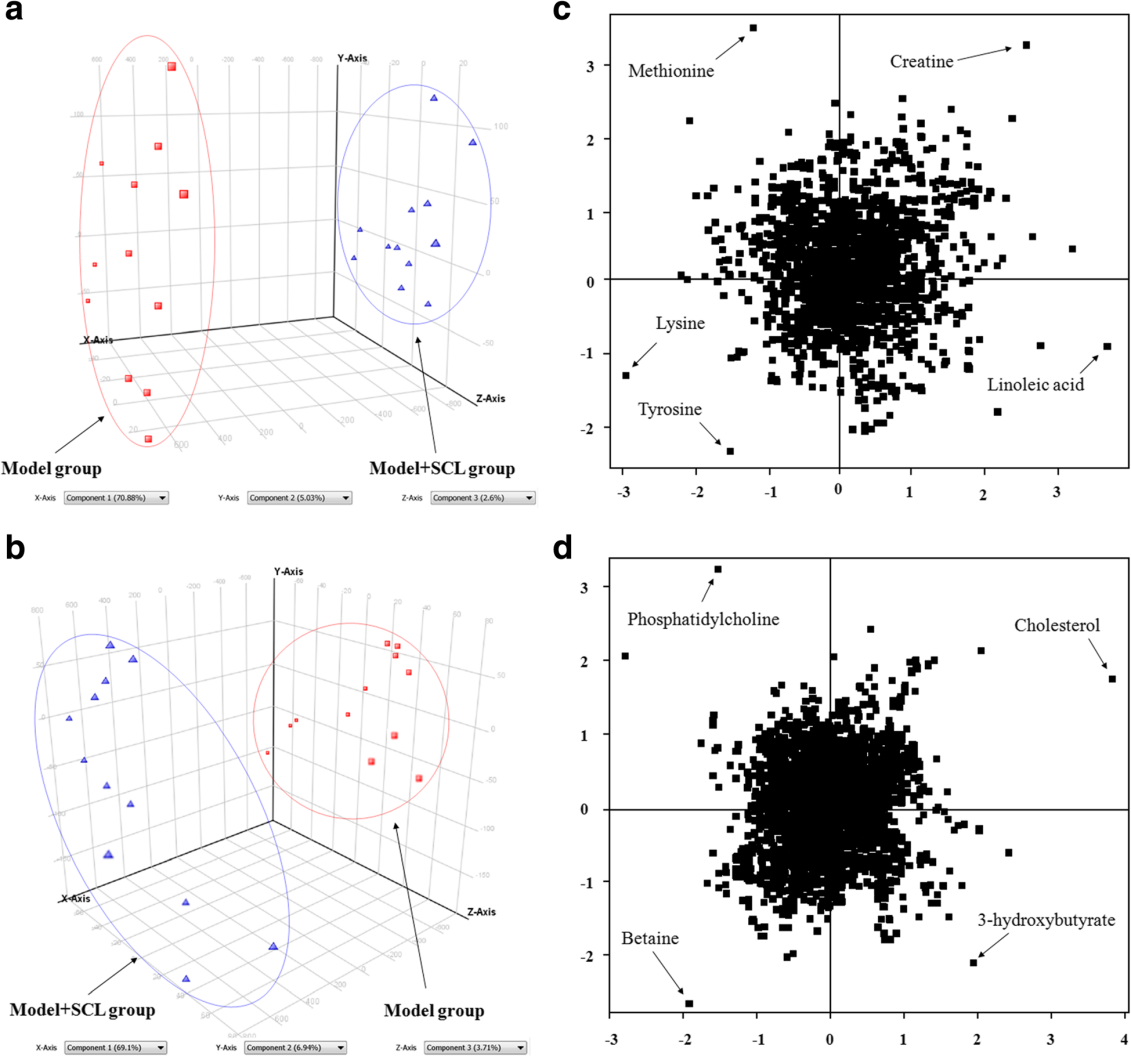
3D–PCA score plots of model and SCL group (: model group; : model + SCL group)in negative (**a**) and positive (**b**) ion mode; loading plots from the result of PCA of model and model + SCL group in negative (**c**) and positive (**d**) ion mode

Based on RRLC-Q-TOF-MS data, the PCA was performed to discover the potential metabolites of serum samples from control, model and SCL mice. PCA, as a common method for multivariate data analysis, can be used to reduce the dimensionality with minimal information loss while retaining the characteristics that contribute most to the variance. Figures 4a-b and 5a-b show the 3D–PCA score plots, in which the scattered points of various samples exhibit an obvious separation in both negative and positive ion modes.

Figures 4c-d and 5c-d show the loading plots from the results of PCA in negative and positive ion modes, and each spot represents one compound. The spots further from the center were considered to make greater contributions to the classification and more likely to be the potential biomarkers. For the biomarkers of the PCA analysis, the PLS-DA method was used to analyze the whole sample data, the R^2^Y and Q^2^ score analysis were 0.937 and 0.879, respectively. The results showed that the validity of current model. Variables important for the projection (VIP) in PLS-DA were used to confirm the potential biomarkers with the VIP values ≥1.

Accordingly, there were 13 potential metabolites which showed significant differences between control and model groups, and 9 of them showed significant differences between model and SCL groups. To identify these potential biomarkers, the retention time, accurate molecular ion mass and characteristic MS/MS fragment ions were compared with those of authentic standards and database resources. Table 2 lists the identified biomarkers and the related information.

**Table 2:** Data of identified metabolites detected by RRLC-Q-TOF/MS

Ion mode	Metabolite	Mass	Molecular formula	Related pathway	Changes (M-C)	Changes (S-M)
Negative	Lysine	146.1876	C_6_H_14_N_2_O_2_	Amino acid metabolism	**↓**	**↑**
	Creatine	113.1179	C_4_H_7_N_3_O	Energy metabolism	**↓**	**↑**
	Methionine	149.2113	C_5_H_11_NO_2_S	Amino acid metabolism	**↓**	**↑**
	Glutamine	146.1445	C_5_H_10_N_2_O_3_	Amino acid metabolism	**↓**	**—**
	Isoleucine	131.1729	C_6_H_13_NO_2_	Amino acid metabolism	**↓**	**—**
	Linoleic acid	280.4455	C_18_H_32_O_2_	Fatty acid metabolism	**↓**	**↑**
	Tyrosine	181.1885	C_9_H_11_NO_3_	Amino acid metabolism	**↓**	**↑**
	Valine	117.1463	C_5_H_11_NO_2_	Amino acid metabolism	**↓**	**—**
	Citric acid	192.1235	C_6_H_8_O_7_	TCA cycle	**↓**	**—**
Positive	Cholesterol	386.6535	C_27_H_46_O	Fatty acid metabolism	**↑**	**↓**
	3-hydroxybutyrate	104.1045	C_4_H_8_O_3_	Lipid metabolism	**↓**	**↑**
	Betaine	117.1463	C_5_H_11_NO_2_	Amino acid metabolism	**↑**	**↓**
	Phosphatidylcholine	758.0603	C_42_H_80_NO_8_P	Lipid metabolism	**↓**	**↑**

Note: M-C represented hyperlipidemia model group vs. control group; S-M represented SCL group vs. hyperlipidemia model group. “**↑**” and “**↓**” indicated that the compound was up- and down-regulated; “**—**” indicated that the compound did not significantly change

**Fig. 6:**
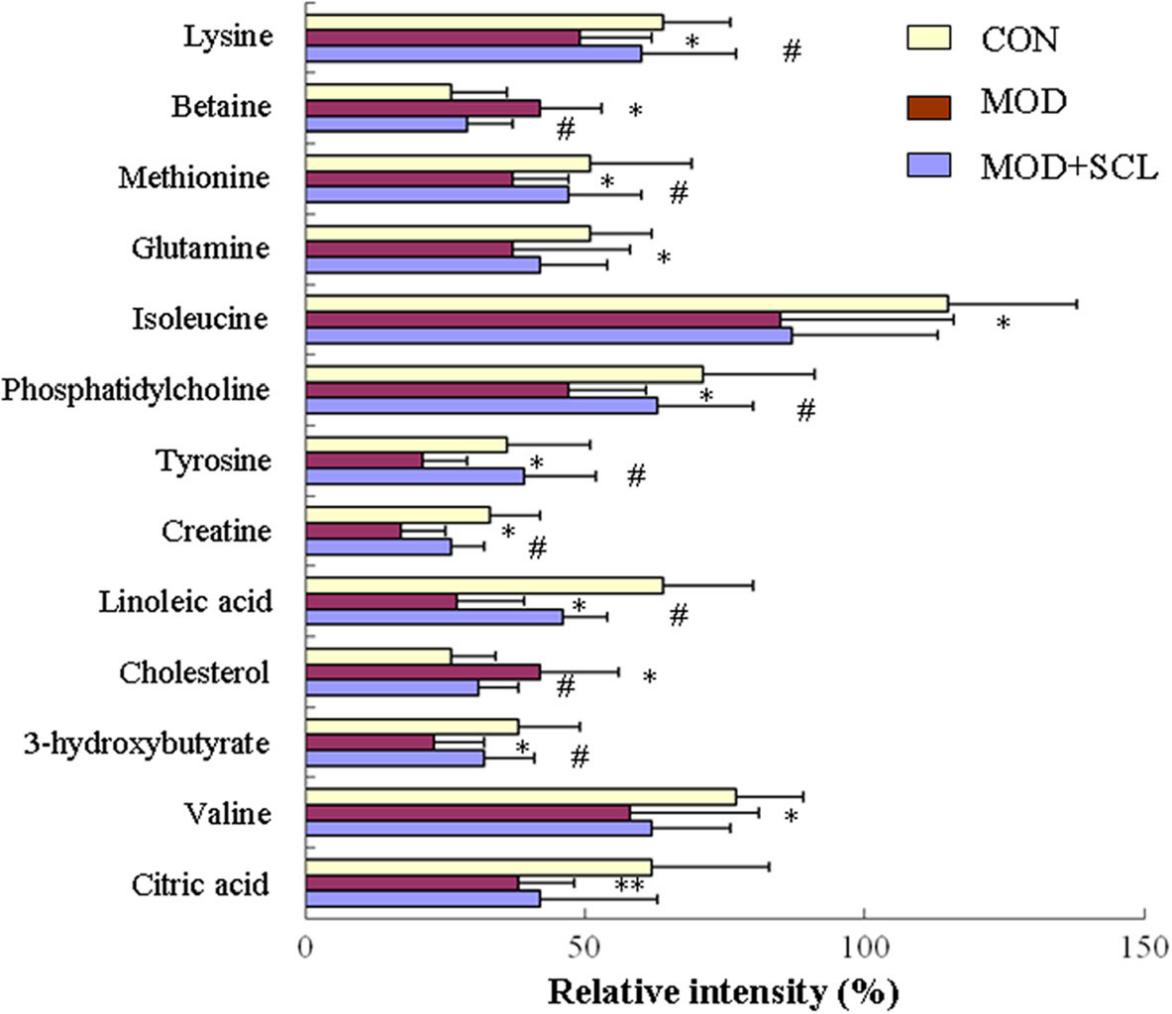
Comparison of the relative intensity of potential biomarkers in control, model and model + SCL group. Note: Values are expressed as mean ± standard deviation (SD). *: *P* < 0.05 and **: *P* < 0.01 compared with the control group; ^#^: *P* < 0.05 compared with the model group

To observe the trends of these metabolites in each group, the extracted ion chromatogram (EIC) and the average peak area of every biomarker of respective groups were analyzed to achieve the specific peak information for the relative intensity. The peak area of all the metabolites in each group was presented as the mean ± SD. The statistical analysis was performed by ANOVA and the differences between the means were assessed by Turkey’s test. The statistical significance was considered as the value of *P* < 0.05 and *P* < 0.01. Figure 6 shows the changes of biomarkers in each group.

### Effect of SCL on the expression of SREBPs and its related protein genes

**Fig. 7:**
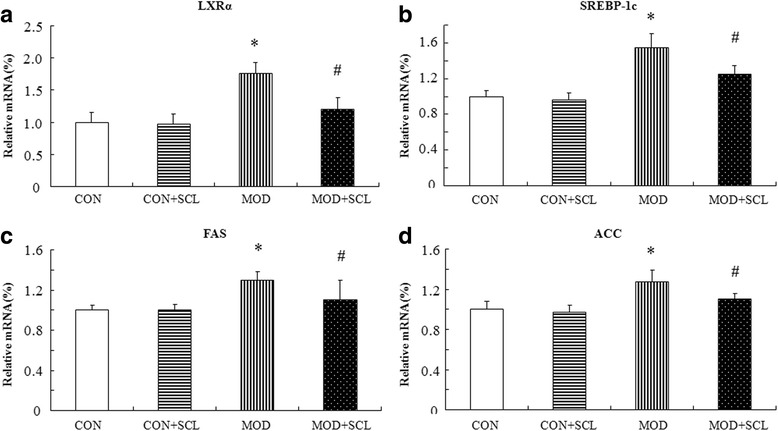
Effects of SCL on the mRNA expression of lipid synthesis and its related genes. Note: *: *P* < 0.05 compared with those in the control group; ^#^: *P* < 0.05 compared with those in the model group

**Fig. 8:**
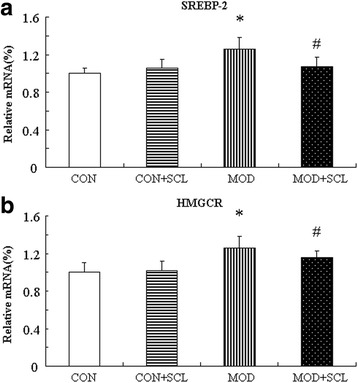
Effects of SCL on the mRNA expression of cholesteral synthesis and its related genes. Note: *: *P* < 0.05 compared with the control group; ^#^: *P* < 0.05 compared with those in the model group

As shown in Fig. 7a-d, compared with those in the control group, the key regulator of liver lipid synthesis SREBP-1c and its related gene FAS/ACC expression levels were elevated in the liver of hyperlipidemia mice (*P* < 0.05). Compared with those in the model group, their expression levels were significantly inhibited in the SCL-treated mice (*P* < 0.05), and the expression of hepatic SREBP-1c upstream gene LXR_α_ was also significantly inhibited (*P* < 0.05). At the same time, the key regulators of hepatic cholesterol synthesis SREBP-2 and HMGCR expressions were also significantly decreased in the SCL-treated mice (*P* < 0.05), as shown in Fig. 8a-b. The expression level of SREBPs and its related protein genes between the control group and control + SCL group were not significantly different (*P* > 0.05).

## Discussions

Feeding HFD is the most commonly method for animal model of hyperlipidemia [25]. In this study, the serum TC and TG level showed an obviously increase in hyperlipidemia model group after HFD feeding for 4 weeks compared with that in the control group (*P* < 0.01), and 4 weeks after administration of SCL, the serum TC (*P* < 0.05), TG (*P* < 0.01), and LDL-c (*P* < 0.01) were significantly decreased, whereas the serum HDL-c (*P* < 0.01) was significantly increased in SCL group compared with that in the hyperlipidemia model group, but the serum TC, TG, LDL-c and HDL-c levels between the control group and control + SCL group were not significantly different (*P* > 0.05). These changes in the serum biochemical indexes showed that the mouse should have developed a metabolic disorder of lipids and/or lipoproteins in the serum. This indicated that the HFD-induced hyperlipidemia model had been successfully established in the mouse after a continuous administration of a HFD for 4 weeks and SCL could have a significant effect on lipid metabolism disorders, but no effects on normal mice, which was also verified by the metabolomics analysis in the scores plot.

The study demonstrated that the levels of lysine, methionine, isoleucine, phosphatidylcholine, tyrosine, creatine, linoleic acid, glutamine, 3-hydroxybutyrate, valine and citric acid decreased remarkably, while the levels of betaine and cholesterol increased obviously in serum of hyperlipidemia model mice compared to those in the control group. These change trends indicate that hyperlipidemia is closely related to energy metabolism, lipid metabolism and amino acid metabolism.

### Metabolites related to energy metabolism

Creatine is a commonly used as an indicator of renal function, the creatine-phosphocreatine system is crucial for mitochondria transport energy production, and the decrease level of creatine in hyperlipidemia model group can taken as a disruption signal of energy homeostasis [26]. Recently metabolomic studies have reported that the reduced level of serum creatine is related to steatosis and non-alcoholic fatty liver disease [27–29]. Citric acid, as one of the important intermediates in the tricarboxylic acid cycle (TCA cycle), is involved in the physiological oxidation of fats, proteins, and carbohydrates to carbon dioxide and water, a decreased level of citric acid also indicates a glycolysis inhibition and energy metabolism dysfunction in hyperlipidemia model mice [30]. In this study, the levels of creatine (*P* < 0.05) and citric acid (*P* < 0.01) decreased obviously in the model group compared with that in the control group, while the creatine level in model + SCL group (*P* < 0.05) increased obviously compared with that in the model group. These results suggest that SCL could prevent the renal damage caused by lipid disorders.

### Metabolites related to amino acids

Amino acids, as substrates for protein synthesis, gluconeogenesis and ketogenesis, can also provide energy through TCA cycle [31, 32]. Valine, isoleucine and tyrosine are branched chain amino acids, playing an important role in promoting protein synthesis, glucose metabolism, oxidation and regulating leptin secretion. High levels of them contribute to obesity-related insulin resistance and glucose intolerance [33–35]. Isoleucine and valine are also glucogenic amino acids. The decrease in these amino acids indicates that they should generate α-keto acid through deamination, and α-keto acid subsequently generates glucose via gluconeogenesis [36]. Tyrosine is an essential amino acid and the precursor to catecholamines, such as epinephrine, dopamine and norepinephrine [37]. Epinephrine has shown a role to adjust phospholipid metabolism, and lipid distribution and transport in serum [38]. Many studies indicated that tyrosine might be a potential biomarker for hyperlipidemia [39]. In this study, the level of valine (*P* < 0.05), isoleucine (*P* < 0.05) and tyrosine (*P* < 0.05) decreased obviously in the model group compared with that in the control group, while the level of tyrosine (*P* < 0.05) in model + SCL group increased obviously compared with that in the model group. These results indicate the regulation of SCL on lipid metabolism through epinephrine pathway by evaluating the tyrosine level.

Glutamine is the precursor of glutathione, which is an important antioxidant, and the lower level of glutamine indirectly may lead to increased lipid peroxidation damage. Previous studies in many patients found that serum glutamine concentrations could be an important signal during the pathogenesis of metabolic diseases [40]. In this study, the level of glutamine (*P* < 0.05) decreased obviously in the model group compared with that in the control group, and there was no significant difference between model group and model + SCL group.

The lysines in albumin- and apoB100- containing particles can generate glycosylation end products or can be further oxidized [41, 42], thus the lower free lysine level in hyperlipidemia group might indicate the occurrence of oxidative stress in the pathogenesis of hyperlipidemia, which might aggravate fatty liver disease and fat accumulation. The level of lysine (*P* < 0.05) decreased obviously in the model group compared with that in the control group, while SCL administration caused the increase of lysines level (*P* < 0.05) compared with the model group in our study. This result may implicate that anti-oxidative activity should be one of the possible properties of the SCL.

Methionine is an intermediate in a transmethylation reaction that uses S-adenosyl methionine as a methyl donor to homocysteine [43]. By acting as a methyl donor during the remethylation of homocysteine, betaine converts homocysteine into methionine and helps maintain the appropriate level of S-adenosyl methionine. Betaine was discovered to be the most important variable in predicting hyperlipidemia, and could be considered an early biomarker of coronary heart disease [44, 45]. Our study showed a lower level of methionine (*P* < 0.05) while higher level betaine (*P* < 0.05) in hyperlipidemia group, and the levels of methionine and betaine returned to normal levels after SCL administration. These results suggest that SCL can regulate the methionine/homocysteine cycle enhancement of hyperlipidemia and reduce the risk of cardiovascular diseases.

### Metabolites related to lipid metabolism

Lipid metabolism disorder is the most obvious manifestation of the development of hyperlipedemia. Phosphatidylcholine, as the most predominant lipid in the HDL fraction [46], can affect the deposition of lipids and cholesterol by removing excessive triglycerides and improving the solubility of cholesterol and lipids in the serum [36]. In this study, phosphatidylcholine level showed an obviously decrease in model group serum compared with that in the control group (*P* < 0.05), and an increase in SCL group compared with that in the model group. These changes were consistent with the results of HDL-c level in serum.

Linoleic acid is an essential fatty acid and exclusively reflects the dietary intake [47]. Recent research has reported linoleic plays an important role in reducing the serum TC and LDL-c levels [48]. As a polyunsaturated fatty acid, linoleic acid is a precursor of prostaglandins via the arachidonic acid pathway. Prostaglandins have many beneficial effects against cardiovascular risk, including hyperlipidemia and essential hypertension [49, 50]. The level of linoleic acid decreased (*P* < 0.05) obviously in the model group compared with that in the control group, while SCL administration caused an increase in linoleic acid level (*P* < 0.05) compared with model group in our study. According to the experimental results of serum TC and LDL-c, this result indicates that SCL can regulate TC and LDL-c disorder through linoleic acid pathway.

Cholesterol is an important biomarker of hyperlipidemia, and its high serum level in hyperlipedemia model group (*P* < 0.05) and its returning to the normal level after SCL administration (*P* < 0.05) in this metabolism study were consistent with those by the serum lipid measurement.

3-hydroxybutyrate is generally considered to be ketone body, produced by aceoacetate and acetyl-CoA. It was previously reported that the decrease in 3-hydroxybutyrate demonstrated that hyperlipidemia could lead to the accumulation of ketone body and the conversion of acetoacetate transfers towards the production of acetone [36]. The level of 3-hydroxybutyrate decreased (*P* < 0.05) obviously in the model group compared with that in the control group, while SCL administration caused an increased 3-hydroxybutyrate level (*P* < 0.05) compared with the model group in our study, indicating that SCL can regulate acetyl-CoA to restore the disorder of hyperlipedemia metabolism.

**Fig. 9:**
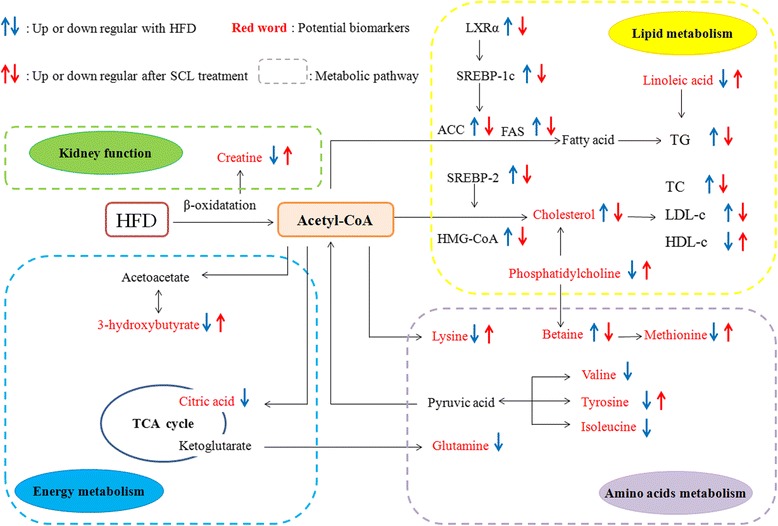
Potential metabolic pathways disturbed in hyperglycemia mice induced by HFD and alterations by SCL treatment. Notes: “” and “” in blue indicate that in HFD model group up- and down-regulated compared with the control group; “” and “” in red indicate that in model + SCL group up- and down-regulated compared with the HFD model group

In summary, SCL treatment partially recovered the metabolism disorders induced by high-lipid diet and exerted a good anti-hyperlipidemia effect. The metabolic pathways included were proposed as follows: TCA cycle, synthesis of ketone body and cholesterol, choline metabolism, and fatty acid metabolism (Fig. 9).

### Effects of SCL on the mRNA expression of lipid/cholesterol metabolism-related genes in hyperlipidemic mice

Liver is an important organ of regulating lipid metabolism balance. The key enzymes of liver fatty acid synthesis, acetyl-CoA carboxylase (ACC) and fatty acid synthase (FAS), are regulated by the nuclear transcription factor SREBP-1c [51]. The related experiments have confirmed that in transgenic mice, ob/ob mice (the insulin resistance and hyperinsulinemia caused by leptin deficiency), mice and rats on diets containing higher cholesterol, other hyperlipidemia animals models, and patients with high blood lipids, the expressions of SREBP-1c and its lipid synthesis-regulated target genes in the livers, including FAS and ACC expression, were increased several times, resulting in a large number of TG deposition [52–56], and the gene expression of SREBP-1c is regulated by the nuclear liver X-activated receptor-α (LXRα). The activation of LXR can increase the expression of SREBP-1c, FAS and ACC genes, causing an increase in the content ratio of TG [57]. In addition, the expression of LXRα/SREBP-1c is significantly increased in animals and patients with hyperlipidemia, showing a positive correlation [58]. Consistent with these findings, our results indicated that the expressions of LXRα, SREBP-1 and its target genes, and ACC and FAS, were significantly increased in the liver tissues of hyperlipidemia mice on the high-fat diet for 4 weeks, while the administration of SCL could down-regulate the expression of LXRα/SREBP-1c signal, and then reduce the expression of FAS and ACC, thereby reducing the production of TG and fatty acid and the lipid accumulation in the liver.

Moreover, we found that the administration of SCL could also significantly decrease the expression of hepatic cholesterol metabolism key regulatory factors SREBP-2 and HMGCR in hyperlipidemia mice. In recent years, besides fatty acids, the abnormality of cholesterol metabolism and its toxicities in hyperlipidemia, especially the important role in the pathogenesis of NASH, have aroused a wide concern of many researchers [59]. Puri, et al. found that the free cholesterol level in the liver of patients with NASH increased significantly [60]. Then Caballero, et al. confirmed that not only free cholesterol but also SREBP-2 levels were also significantly increased in patients with hyperlipidemia [61]. Furthermore, it is well known that the excessive free cholesterol can be oxidized to oxidized cholesterol, and the oxidized cholesterol can up-regulate the synthesis of fatty acids and triglycerides by activating LXRα/SREBP-1c pathway, further promoting the accumulation of fat [62–64]. In this study, the expression of SREBP-2 and its target genes HMGCR was significantly increased in the liver of mice with hyperlipidemia induced by HFD and the administration of SCL could decrease the expression of key factors of cholesterol synthesis, reduce the cholesterol synthesis and alleviate the toxicity of cholesterol in the mice.

## Conclusions

In the present work, anti-hyperlipidemia effect of *Schisandra Chinensis* lignans was confirmed by both serum biochemistry and metabolomics analysis. We employed RRLC-Q-TOF-MS-based metabolomics approaches to reveal metabolism regulations of SCL on hyperlipidemia mice induced by HFD feeding. It turned out that the administration of SCL could partially recover the metabolism dysfunction caused by hyperlipidemia via the possible metabolic pathways: TCA cycle, synthesis of ketone body and cholesterol, choline metabolism and fatty acid metabolism. The mechanism may be related to the down-regulation of LXRα/SREBP-1c/FAS/ACC and SREBP2/HMGCR signaling pathways, which may provide a theoretical basis for the prevention and treatment of hyperlipidemia.

## Abbreviations

ACC: acetyl-CoA carboxylase
FAS: fatty acid synthase
HDL-c: high-density lipoprotein cholesterol
HFD: high-fat diet
HMGCR: 3-hydroxy-3-methylglutaryl coenzyme A reductase.
KEGG: Kyoto Encyclopedia of Genes and Genomes
LDL-c: low-density lipoprotein cholesterol
LXRα: liver X receptor α
PCA: principal component analysis
PLS-DA: partial least squares-discriminant analysis
RRLC-Q-TOF-MS: rapid resolution liquid chromatography coupled with quadruple-time-of-flight mass spectrometry
RT-PCR: real-time polymerase chain reaction
SCL: *Schisandra Chinensis* lignans
SREBPs: sterol regulatory element-binding proteins
TC: total cholesterol
TCA: tricarboxylic acid
TG: triglyceride
